# Efficacy of TENS, ultrasound and low level laser in the management of TMJ disorder

**DOI:** 10.6026/973206300210969

**Published:** 2025-05-31

**Authors:** Shreedevi Bhoi1, Diksha Koushal, Abhigyan Manas, Ruhi Sidhu, Navdeep Kaur Shergill, Ankur Kakkad, Kiran Kumar Misra, Awadhesh Kumar Gupta

**Affiliations:** 1 Department of Oral and Maxillofacial Surgery, ESIC Dental College and Hospital, Kalaburgi, Karnataka, India; 2 Department of Oral Medicine and Radiology, Jaipur Dental College, Jaipur, Rajasthan, India; 3 Department of Oral & Maxillofacial Surgery, Uttar Pradesh University of Medical Sciences, Saifai, Etawah, Uttar Pradesh, India; 4 Department of Oral Medicine and Radiology, MNDAV Dental College and Research Centre, Solan, Himachal Pradesh, India; 5 Department of Oral Medicine and Radiology, Range Imaging Centre, Gurgaon, Haryana, India; 6 Department of Oral Medicine and Radiology, Hitkarini Dental College and Hospital, Jabalpur, Madhya Pradesh, India; 7 Department of Oral and Maxillofacial Surgery, Kalinga Institute of Dental Sciences, KIIT (Deemed to be University), Patia, Bhubaneswar, Odisha, India; 8 Department of Oral Pathology and Microbiology, DJ College of Dental Sciences and Research, Modinagar, Ghaziabad, Uttar Pradesh, India

**Keywords:** Low level laser, management, photo-bio-modulation, transcutaneous electrical nerve stimulation (TENS), temporomandibular joint (TMJ) disorder

## Abstract

Conservative treatments for temporomandibular disorders (TMD) are pain alleviation and the restoration of normal jaw function. Therefore, it is of interest to assess the transcutaneous electrical nerve stimulation (TENS), laser and ultrasound therapy in management of temporomandibular disorders. Hence, a total of 36 patients with TMJ disorder were divided into 3 groups; TENS, low level laser and ultrasound. The treatment consists of 10 sessions over the course of four weeks. The efficacy of treatment was evaluated using clinical mouth opening, muscle tenderness and visual analogue scale (VAS) for pain. The efficacy of TMD management is similar with all three methods.

## Background:

The masticatory muscles, temporomandibular joint (TMJ), and head and neck musculature are all affected by Temporomandibular Disorders (TMD), which are musculoskeletal disorders of the masticatory system [1, 2]. The illness known as temporomandibular dysfunction (TMD) affects how the jaw moves. It is brought on by a number of clinical issues involving the myofascial muscles, temporomandibular joint (TMJ), and other structures that can have a major impact on a patient's quality of life [3]. The incidence of TMD is 13% in adults and 11% in children [4]. Numerous clinical signs and symptoms, including discomfort during chewing, neck pain, headaches, clicking, tinnitus, TMJ sounds, restricted jaw mobility, deviation, locked jaw, pain in the oral or myofascial masticatory muscles, and aberrant jaw movement, are indicative of a multifactorial aetiology. The masticatory musculature becomes tenser as a result of symptoms in most cases [5, 6]. Temporomandibular disorder (TMD)-related pain is prevalent issues that can have a major impact on a patient's quality of life [7]. In order to effectively manage temporomandibular joint disorders (TMDs), a number of therapeutic modalities have been recommended [2]. Physiotherapy, manual therapy, occlusal splints, laser therapy, oral and injectable medication, and in more extreme situations, surgery, are all options for treating TMD. Occlusal splints, exercises, and massage therapy are the most regularly reported treatments and are frequently effective [8, 9]. Both invasive and non-invasive techniques are used in treatment. TENS, LLLT, ultrasound, acupuncture, TMJ mobilisation, and manual treatments are among the physical therapy interventions that have been identified to have the potential to be beneficial. Furthermore, there are a number of non-surgical therapeutic alternatives, including relaxation techniques, detachable gadgets, and physical therapy [10, 11-12]. In recent years, laser therapy has emerged as an additional dental treatment. Due to their non-invasive treatment qualities, lasers are becoming more and more popular in the treatment TMD, offering patients a potential treatment option for the condition in the future. Numerous medical specialities have employed laser therapy, most especially photobiomodulation (PBM) using varying laser wavelengths, to reduce pain and regulate inflammation [13]. According to reports, laser photobiomodulation (PBM) is a potential medical technique for treating temporomandibularjoint (TMJ) discomfort, wound and bone healing, and more [7]. Prior research on the use of 1064 Nd:YAG lasers demonstrated that they were just as successful in reducing pain in patients with myofascial pain dysfunction syndrome as occlusal splints [14]. Therefore, it is of interest to assess the transcutaneous electrical nerve stimulation (TENS), low level laser and ultrasound therapy in management of temporomandibular disorders.

## Materials and Methods:

Total 36 individuals (20 men and 16 women) participated in this prospective, randomised clinical trial at the Department of Oral Medicine. The institutional ethical review board gave its approval to the study protocol. The participants had a clinical diagnosis of TMD linked to clicking joint sound, pain in the masticatory muscles, chronic TMD of muscular origin, temporomandibular joint (TMJ) pain, and limited mouth opening. The study did not include patients with heart pacemakers, cancer, or any other serious systemic disease. The study was preceded with investigations such as a complete blood count, clotting time, orthopantomography (OPG), and cone beam computed tomography (CBCT). An investigator with training conducted the study. Three groups of participants were haphazardlyallocated to receive treatment: Group I received TENS, Group II received low-level laser beam therapy, and Group III received ultrasound therapies. For 30 days, ten treatments of nine minutes each on each side of the face were used to treat the condition using the appropriate technique. A low-level laser device (VRkc-610 SOFT LASER - Dentoflex, São Paulo -SP, Brazil) with a wavelength of 830 to 904 nm and an output of 4J/cm2 and 100mW was utilised. The low level laser therapy was administered to the LASER group using "scanning" motions rather than direct skin contact over the sore area. Together with surface electrodes Myo-trodes II (Myotronics, Seattle, USA), the AalayMedlnsTM Mini Stimulator was chosen for transcutaneous electric stimulation (TENS). It has an output frequency of 2-130 Hz and rhythmically delivers a low level electric pulse every 1. 5 seconds. The third group was exposed to ultrasound (Manual US Mini, SAS 180, Delhi) for 10 minutes at an output of 1.0 W/cm^2^. Pain-free mouth opening and the Visual Analogue Scale (VAS) score were evaluated. The mouth openness was measured both during and after therapy using a verniercalliper. The VAS is administered using a 100mm horizontal line with the words "no pain" at the left end and "the worst possible pain" at the right.

## Palpation of muscle sites:

All of the masticatory muscles-the masseter, lateral, medial, and temporalis muscles-were palpated using the conventional palpation technique. Digital palpation was used to record the presence or absence of muscular soreness. Bilateral muscle palpation was carried out using a steady, firm, and moderate pressure of about 1500 grammes [1]. The obtained data were statistically analysed using Analysis of Variance (ANOVA), t-test and post-hoc Tukey test.

## Results:

Intra group comparison was highly significant for VAS pain score from before and after treatment (Table 1). Table 2 indicates that there was improvement in muscle tenderness for all 3 groups from baseline to after 4 weeks of treatment. LLT group showed more effective compared to other group but it was statistically insignificant. Intra group comparison was highly significant for mouth opening score from before and after treatment. There was increase in mouth opening in mm after therapy. Group I was most effective compared to other groups (Table 3).

## Discussion:

A combination of the mandibular condyle and its related temporal cavity components, the articular eminence and glenoid fossa, structurally constitute the temporomandibular joint (TMJ), one of the body's most remarkably complex synovial joints [4, 15]. The term "temporomandibular disorders" (TMDs) refers to a group of clinical issues that affect the temporomandibular joint (TMJ), the masticatory muscles, related tissues, or both [15, 16-17]. According to the current study, TMD patients' mouth opening, muscular soreness, and discomfort were all improved by the three non-invasive treatments TENS, LLLT, and ultrasound. When it came to pain relief, LLLT outperformed TENS therapy. But when it came to enhancing the mouth opening, TENS worked better than any other technique. According to Regulski *et al.* case report, PBM combined with a Nd:YAG laser may be a useful tool for treating orofacial discomfort in individuals who have both acute and chronic TMJ disc displacements. It may also shorten recovery times [7]. In their evaluation of the efficacy of TENS, LLLT and ultrasound therapy in treating TMDs, Yeladandi *et al.* discovered that all three groups experienced improvements in mouth opening and decreases in VAS scores [2]. TENS, ultrasound, and low-level laser treatment (LLLT) were reported to be successful in treating TMD by Darwin *et al.* [4]. The effectiveness of TENS and laser therapy in treating TMJ issues was evaluated by Kato *et al.* The visual analogue scale (VAS) of pain and active range of motion (AROM) were used to evaluate the effectiveness, and the results showed that both treatments are useful in the treatment of TMD [1]. Our findings are consistent with the outcomes of previous investigations. In their evaluation of the efficacy of TENS treatment and LLLT in treating temporomandibular joint (TMJ) disorders, Mishra *et al.* came to the conclusion that LLLT seemed to perform better than TENS therapy in terms of muscle tenderness variables [18]. This has to do with our outcome. Right after the irradiation, PBMT was demonstrated to be successful in lowering orofacial/cervical skull discomfort [19]. Anupriya came to the conclusion that while both LLLT and TENS had positive outcomes, LLLT appeared to be marginally more effective than TENS therapy in terms of measuring muscle tenderness characteristics [17]. Brochado *et al.* evaluated the efficacy of manual treatment (MT) and photobiomodulation (PBM), either separately or in combination (CT), and came to the conclusion that all of the tested techniques improved mandibular function and reduced pain [20]. According to a systematic review by Farshidfar *et al.* PBMT had encouraging results on pain relief and MMO improvement [21]. Ansari *et al.* from systemic review concluded that, TENS, LLLT, and US helps in management of TMD [22]. In addition to being linked to temporomandibular disorders, discomfort in the TMJ area should also be distinguished from other illnesses. Primary neuralgias are primarily linked to the sharp pain. Surgery may be necessary for TMJ issues if conservative therapy approaches prove ineffective [7]. The current study's limitation is its one-month follow-up duration, which was also carried out on a small sample. None of the individuals in this study reported experiencing any negative side effects during or after treatment.

## Conclusion:

Transcutaneous electrical nerve stimulation, low level laser and ultrasound treatments were equally successful in managing temporomandibular disorders.

## Figures and Tables

**Table 1:** VAS score changes before and after treatment are compared

**Treatment Group**	**VAS score**		**Mean difference ±SD**	**Standard error**	**p**
	**Before**	**After**			
Group 1: TENS	5. 74±1. 22	2. 71±0. 76	3. 03±0. 46	0. 28	0. 001
Group 2: LLT	5. 87±1. 28	1. 20±0. 53	4. 62±0. 75	0. 37	0. 001
Group 3: Ultrasound	6. 34±1. 34	1. 25±0. 46	5. 14±0. 88	0. 46	0. 001

**Table 2:** Evaluation for muscle tenderness among the groups

**Treatment Group**	**Muscle tenderness**		**Mean difference ±SD**	**Standard error**	**p**
	**Before**	**After**			
Group 1: TENS	1. 02±0. 0	0. 82±0. 42	0. 2±0. 42	0. 14	0. 001
Group 2: LLT	1. 02±0. 0	0. 43±0. 32	0. 59±0. 32	0. 34	0. 001
Group 3: Ultrasound	1. 02±0. 0	0. 62±0. 21	0. 4±0. 21	0. 39	0. 001

**Table 3:** Mean mouth opening (MMO) changes before and after treatment are compared

**Treatment Group**	**Mouth opening (mm)**		**Mean difference ± SD**	**Standard error**	**p**
	**Before**	**After**			
Group 1: TENS	26. 53±3. 53	34. 62±4. 65	8. 09±1. 07	0. 76	0. 001
Group 2: LLT	25. 25±3. 65	32. 15±4. 43	6. 90±0. 78	0. 98	0. 001
Group 3: Ultrasound	26. 65±3. 34	31. 21±4. 76	4. 61±1. 42	0. 65	0. 001
P	0. 876	0. 001			
